# A Multidisciplinary Update on Treatment Modalities for Metastatic Spinal Tumors with a Surgical Emphasis: A Literature Review and Evaluation of the Role of Artificial Intelligence

**DOI:** 10.3390/cancers16162800

**Published:** 2024-08-08

**Authors:** Rebecca Houston, Shivum Desai, Ariel Takayanagi, Christina Quynh Thu Tran, Ali Mortezaei, Alireza Oladaskari, Arman Sourani, Imran Siddiqi, Behnood Khodayari, Allen Ho, Omid Hariri

**Affiliations:** 1 Department of Neurosurgery, Arrowhead Regional Medical Center, 400 N Pepper Ave, Colton, CA 92324, USA; omid.r.hariri@kp.org; 2 Department of Neurosurgery, Ascension Providence Hospital, 16001 W Nine Mile Rd, Southfield, MI 48075, USA; shivum.desai@midwestern.edu; 3 Department of Neurosurgery, Riverside University Health System, 26520 Cactus Ave, Moreno Valley, CA 92555, USA; ariel.takayanagi@tu.edu (A.T.); imran.siddiqi@westernu.edu (I.S.); 4 Kaiser Permanente Bernard J. Tyson School of Medicine, 98 S Los Robles Ave, Pasadena, CA 91101, USA; christina.q.tran@kp.org; 5 Student Research Committee, Gonabad University of Medical Sciences, Gonabad 9P67+R29, Razavi Khorasan, Iran; mortezaei.a.stu@gmu.ac.ir; 6 School of Biological Sciences, University of California Irvine, 402 Physical Sciences Quad, Irvine, CA 92697, USA; aoladask@uci.edu; 7 Department of Neurosurgery, Isfahan University of Medical Sciences, Hezar Jerib Avenue, Isfahan JM76+5M3, Isfahan, Iran; armansourani@resident.mui.ac.ir; 8 Environment Research Center, Research Institute for Primordial Prevention of Non-Communicable Disease, Isfahan University of Medical Sciences, Hezar Jerib Avenue, Isfahan JM76+5M3, Isfahan, Iran; 9 Department of Radiation Oncology, Kaiser Permanente Los Angeles Medical Center, 4867 W Sunset Blvd, Los Angeles, CA 90027, USA; behnood.khodayari@kp.org; 10 Department of Neurological Surgery, Kaiser Permanente Orange County, 3440 E La Palma Ave, Anaheim, CA 92806, USA; allen.l.ho@kp.org; 11 Department of Surgery, Western University of Health Sciences, 309 E 2nd St, Pomona, CA 91766, USA; 12 Department of Orthopedic Surgery, University of California Irvine School of Medicine, 1001 Health Sciences Rd, Irvine, CA 92617, USA

**Keywords:** spinal metastasis, carbon-fiber-reinforced poly-ether-ether-ketone, minimally invasive surgery, next-generation sequencing, artificial intelligence, neurosurgery

## Abstract

**Simple Summary:**

As the treatment modalities for spinal metastatic tumors continue to evolve, the goal of improving the quality of life of patients with spinal metastases becomes more easily attainable. The best patient care is a combination of multiple treatment modalities, surgical and nonsurgical, that maximizes the advantages of each modality and should be individualized according to their unique clinical presentation, pathology, and life expectancy. In this review article, we highlight various new treatment options that have shown promising improvements in patient outcomes. Notably, we discuss the potential clinical applications of AI and NGS in the treatment of spinal metastases.

**Abstract:**

Spinal metastases occur in up to 40% of patients with cancer. Of these cases, 10% become symptomatic. The reported incidence of spinal metastases has increased in recent years due to innovations in imaging modalities and oncological treatments. As the incidence of spinal metastases rises, so does the demand for improved treatments and treatment algorithms, which now emphasize greater multidisciplinary collaboration and are increasingly customized per patient. Uniquely, we discuss the potential clinical applications of AI and NGS in the treatment of spinal metastases. Material and Methods: A PubMed search for articles published from 2000 to 2023 regarding spinal metastases and artificial intelligence in healthcare was completed. After screening for relevance, the key findings from each study were summarized in this update. Results: This review summarizes the evidence from studies reporting on treatment modalities for spinal metastases, including minimally invasive surgery (MIS), external beam radiation therapy (EBRT), stereotactic radiosurgery (SRS), CFR-PEEK instrumentation, radiofrequency ablation (RFA), next-generation sequencing (NGS), artificial intelligence, and predictive models.

## 1. Introduction

The reported incidence of spinal metastases has increased in recent years due to innovations in imaging modalities, systemic therapies, and surgical treatments, all of which have led to improvements in the life expectancy of cancer patients [1,2]. Spinal metastases occur in up to 40% of patients with cancer, with 10% of cases ultimately becoming symptomatic [3]. Since local tumor progression can lead to pathological fractures and pain, early detection and treatment of spinal metastases is critical [4]. The goals of treatment for spinal metastasis include local tumor control, improving quality of life, minimizing pain, halting neurological decline, and spinal stabilization [5,6].

Historically, the treatment approach for spinal metastases consisted of posterior decompression alone [7]. With growing evidence suggesting better outcomes with conventional radiotherapy alone, surgical intervention was largely abandoned in favor of conventional radiotherapy as the standard of care [7]. That is, until the 2005 landmark study by Patchell et al., which demonstrated a survival advantage and superior ambulatory outcomes in patients with spinal metastases treated with a combination of surgery and radiotherapy compared to radiotherapy alone [8]. As such, the definitive role of surgical intervention for spinal metastases was established. Strides in surgical approaches and instrumentation have improved patient outcomes. Patients previously considered poor surgical candidates may now benefit from developments in short-segment fixation, cement augmentation, stereotactic radiosurgery, and minimally invasive surgery techniques.

Progress in treatment is naturally followed by an evolution in treatment algorithms, which now emphasize greater multidisciplinary collaboration. By sharing knowledge across disciplines, it can enhance multidisciplinary collaboration and bring benefits such as safer care and improved outcomes for patients. We present a summary of evidence on the current treatment modalities for spinal metastases, written in a way that is easily digestible by nonspecialists and specialists alike.

In this comprehensive review, we provide an update on the current treatment modalities for metastatic spinal tumors. Specifically, we highlight the role of a multidisciplinary team (MDT), minimally invasive surgery (MIS), preoperative embolization, external beam radiation therapy (EBRT), stereotactic radiosurgery (SRS), cement augmentation, fenestrated screws, carbon-fiber-reinforced poly-ether-ether-ketone (CFR-PEEK) instrumentation, and radiofrequency ablation (RFA) in the treatment of spinal metastases. Lastly, we describe the future directions of treatment, emphasizing the role of next-generation sequencing (NGS), artificial intelligence (AI), and predictive modeling in the management of spinal metastases.

## 2. Materials and Methods

A PubMed search was performed for articles regarding the treatment of spinal metastases published in peer-reviewed journals from 1 January 2000 to 31 December 2023. After removing all duplicates, the articles were screened for relevance. The authors then reviewed and selected full-text articles for inclusion. The key findings from each study were synthesized into this report.

## 3. Discussion

### 3.1. Multidisciplinary Team Approach

A multidisciplinary team (MDT) approach to metastatic disease of the spine ensures that patients will receive optimal care during their treatment. Members from spinal surgery, radiation oncology, oncology, pain specialists, nutritional services, psychiatry, and rehabilitative services all play important roles in the treatment of patients with spinal metastases.

Radiation oncology plays a critical role by implementing treatments that are effective in palliating bony pain and providing local tumor control. Rothrock et al. analyzed 1515 patients with spinal metastases over a 20-year period to assess improvements in overall survival [9]. The results demonstrate an increase of 20% in overall survival over the study period, which the authors proposed to be largely due to improvements in chemotherapy, radiation therapy, and immunotherapy [9].

Once a primary neoplasm is identified, oncology plays a pivotal role in evaluating the prognosis and designing a treatment plan. Fortunately, multiple classification systems have been developed, including the Tomita score, the Tokuhashi score (and revised Tokuhashi score), and the NOMS framework, which have all been proven to be effective clinical tools by allowing physicians to efficiently offer individualized care.

Medical pain management can be overseen by primary care or pain management teams but is often managed by oncology. Per the World Health Organization Analgesic Ladder, nonopioid oral analgesia is the first-line therapy for bony pain [10]. Opioids are used for moderate to severe pain, while other medications such as steroids, tricyclic antidepressants, serotonin/norepinephrine reuptake inhibitors, bisphosphonates, Denosumab, and peripheral nerve blocks are also effective in managing pain [11]. Of note, bisphosphonates decrease osteoclastic activity, thereby reducing bone pain and hindering disease progression; these benefits have been shown to be maximized when used in combination with radiotherapy [12]. As the requirements of the patient evolve throughout each stage of treatment, balancing the risks and benefits of pain management may become more difficult. As such, a team familiar with the demands of pain management in the context of oncologic treatment is essential.

The role of a nutritional services team in the management of patients with advanced cancer cannot be overstated, as significant involuntary weight loss can compromise treatment outcomes and patient function [13]. Cancer patients have an increased risk of developing anorexia and cachexia, with resultant poor post-surgical wound healing, reduced immunologic function, and post-radiation complications such as colitis, esophagitis, and reduced GI motility [13,14]. Therefore, screening tests such as the prognostic nutrition index can be used to ensure nutritional goals are met, with a particular focus on vitamin D and calcium supplementation for patients prior to surgery [15].

Psychiatric, rehabilitation, and palliative care support is also extremely important. Up to 50% of patients receiving the life-altering diagnosis of cancer may manifest their distress in the form of anxiety, depression, and other psychiatric conditions [16]. Rehabilitative services, including physical, occupational, and speech therapy, are essential to maximizing patient function and preventing complications such as deep vein thrombosis secondary to venous stasis [17].

Hospital tumor boards consist of healthcare providers from multiple departments that meet regularly to discuss cancer treatment plans. Frequent communication between doctors and surgeons may reduce the number of near-misses, misses, and accidental events throughout the course of a patient’s care [18]. A tumor board can improve communication and coordination of care, leading to increased patient satisfaction and decreased morbidity and mortality [19].

### 3.2. Treatment Approach for Spinal Metastases

Prediction models aid physicians with risk stratification and clinical decision-making. Early models such as the Tomita and Tokuhashi scores were limited by their static nature and inability to incorporate evolving treatments [20]. The revised Tokuhashi score from 2017 improved upon the prognostic ability of the 2005 version by updating the 1-year survival prognosis based on the primary tumor diagnosis [21].

In 2006, Bilsky and Smith introduced the dynamic NOMS (neurologic, oncologic, mechanical, systemic) decision framework, which incorporates these four sentinel decision points to determine the best treatment plan for spine metastases [22]. Neurologic considerations include assessing the degree of spinal cord compromise, myelopathy, and functional radiculopathy [6]. Oncologic assessment is based on the tumor’s expected response and durability of response to available treatments [6]. Mechanical assessment involves determining the extent of mechanical instability, which is defined as the loss of spinal integrity secondary to a neoplastic process that is associated with pain, deformity, and/or neural compromise. Mechanical instability serves as an indication for surgery and can be assessed with the Spinal Instability Neoplastic Score (SINS), which has been shown to reliably predict the need for surgical stabilization [6]. SINS assesses six parameters, including location, pain, alignment, osteolysis, vertebral body collapse, and posterior element involvement [6]. Systemic disease assessment aims to evaluate what treatments a patient can physiologically tolerate and the overall expected patient survival, which is dependent on tumor histology, medical comorbidities, and the extent of the disease [6].

These four assessments help the care team tailor radiotherapy and surgery for each patient’s unique needs. As treatment personalization improves with advances in complex data analysis, physicians must take an increasing number of variables into account. Machine learning (ML), a subset of artificial intelligence, is currently being explored to assist doctors in determining treatment plans and survival predictions, as detailed in the artificial intelligence section.

### 3.3. Minimally Invasive Surgery

Major components of MIS include percutaneous spinal instrumentation, neuronavigation, and tubular and expandable retractors, which facilitate transmuscular approaches [23]. MIS approaches can minimize delays in systemic and radiation therapy by facilitating postoperative recovery and minimizing complications and length of stay [23]. MIS techniques also result in decreased subperiosteal dissection, soft tissue disruption, and blood loss compared to open approaches [24].

Not all patients are candidates for a purely MIS approach. The mini-open approach involves the placement of percutaneous pedicle screws combined with a 1–2-level midline approach for decompression [23]. A matched comparison of mini-open approaches and conventional open surgery resulted in less blood loss and postoperative pain with a mini-open approach while maintaining similar neurological outcomes [23].

### 3.4. Preoperative Embolization

Preoperative embolization is performed before surgery to decrease tumor blood supply, thereby reducing intraoperative blood loss and subsequent blood-loss-related complications [25]. First, a spinal angiogram allows visualization of the vessels supplying the tumor, which are then catheterized and occluded with liquid embolic agents, polyvinyl alcohol particles, gelatin sponges, or embolization coils [26]. A 2012 study by Kato et al. demonstrated a reduction of over 50% in intraoperative blood loss with preoperative embolization, with results showing an average blood loss of 520 mL with embolization and 1128 mL without [27]. A study performed a year later by Kato et al. revealed that surgery should be performed on the same day of preoperative embolization to maximize efficiency [28]. Possible explanations include the recanalization of embolized vessels or the recruitment of collateral flow [28].

In contrast, a 2015 study by Clausen et al. showed that preoperative embolization of spinal metastases did not result in a significant reduction in intraoperative blood loss but did lead to a decrease in operative time (median 90 min vs. 124 min) [29]. Spinal embolization can also provide local tumor control. In a comparison between patients who underwent surgery and stereotactic body radiation therapy (SBRT) with preoperative embolization versus without embolization, Damante et al. found improved local tumor control and pain control in the embolization group (14.2 months vs. 6.3 months) [30].

Although spinal embolization is generally safe, there is still a risk of embolization of the Adamkiewicz artery, which can result in paralysis. A meta-analysis of 37 studies on preoperative embolization of spinal tumors by Griessenauer et al. reported an overall complication rate of 3.1%, with 16.2% of complications resulting in permanent neurological deficits [31]. Overall, the literature supports embolization of spinal tumors as a safe and efficacious adjunct to treatment before surgical intervention for spinal metastases.

### 3.5. External Beam Radiotherapy

While modern advances have allowed us to minimize simultaneous exposure of non-tumor tissue to radiation, the skin and tissue superficial to the tumor will inevitably be radiated. As such, it is recommended that radiation be initiated once the surgical wound is completely healed, which typically takes several weeks following surgery [32,33]. If a poorly healed wound is exposed to radiation, it may lead to wound dehiscence or chronic non-healing wounds [34]. Current research suggests that radiotherapy delivered in the immediate postoperative period of up to 3 days can significantly affect wound healing when compared to postponing postoperative radiation for 3–4 weeks [35]. The general consensus is to wait 2 weeks between surgery and radiation, with a minimum of 7 days [32].

The main types of spine radiotherapy are conventional EBRT, SBRT, and SRS, with the primary difference being the delivery of a higher biological equivalent dose via spine SRS and SBRT compared to EBRT. Conventional EBRT involves the delivery of targeted radiation to the tumor from outside the body and is the standard of care following surgical intervention for most spinal metastases [36]. EBRT is most commonly delivered at a total dose of 30 Gy in 10 fractions [37]. In their review, Witham et al. showed improved neurological function in 36% of patients with MESCC following EBRT alone, while a mean of 17% had worse neurological functioning [38]. A 2017 study by Curtin et al. demonstrated pain reduction in 80% of patients who underwent EBRT, with 30% achieving near-total pain relief [39].

Radiosensitive histologies such as hematologic malignancies and certain solid tumors such as breast, prostate, and ovarian carcinomas are more favorable to conventional EBRT, with the remaining solid tumor types having radioresistant histologies and thus being less amenable to EBRT [37]. Effective treatments for radioresistant spinal metastases will be discussed in the following section.

### 3.6. Stereotactic Radiosurgery

In patients with significant epidural spinal cord compression or mechanical instability, “separation surgery” can be used to create a safe margin around the thecal sac that allows administration of radiation [22]. SRS delivers focused beams of high-dose radiation from many different angles to precisely target spinal metastatic tumors, minimizing radiation of nearby healthy tissue [40]. SRS has been shown to improve local tumor control, neurological function, and duration of response in patients with spinal metastases [40]. In cases where repeat radiation must be administered, SRS can achieve adequate pain palliation and tumor control [41]. Patients in good or excellent clinical condition with severe tumor-related pain can expect to receive the most benefit from SRS treatment [42].

In contrast to conventional EBRT, SRS can improve outcomes independent of tumor histology [40]. It must be noted that Zeng et al. showed poorer local control rates in radioresistant tumors with SRS in comparison to radiosensitive tumors [43]. A prospective study of patients with spinal metastases by Gerszten et al. demonstrated that SRS led to pain improvement in 86% and long-term tumor control in 90% [44]. Of the patients who reported progressive neurologic deficits before treatment, 84% reported clinical improvement after SRS [44]. A well-cited meta-analysis by Kaloostian et al. found an overall local control rate of 92% with SRS. [45]. Overall pain improvement was 83% and ranged as high as 97%, and pain increases following therapy occurred in only 4% of the 1028 included patients [45].

As with any treatment regimen, SRS is not without risk. Pain flare, distinguished from spinal cord injury by its inherently transient onset either during or after treatment, is infrequent but is a possible complication of SRS. In a study of 348 patients treated for spinal metastasis with SRS by Balagamwala et al., the most prevalent reason for radiosurgery was pain (73%) [46]. A total of 73 out of 507 SRS treatments (14.4%) led to the development of pain flare, with predictors of pain flare expansion including higher KPS, female gender, higher prescription dose, and cervical/thoracic tumor location [46]. In the literature, the most commonly reported complications of SRS are toxicity, new-onset neurological deficits, vertebral compression fractures, and worsening of existing neurological deficits [47,48,49].

As such, conventional EBRT remains more commonly used following surgery for spinal metastases. As a result, the decision to pursue treatment with spine SRS versus conventional EBRT should be based on each patient’s clinical circumstances.

### 3.7. Cement Augmentation

Cement augmentation is frequently used for the management of spinal metastases and pathologic compression fractures. In vertebral augmentation, bone cement is injected through the skin into fractured vertebrae to fill lytic cavities and restore vertebral body height, resulting in decreased pain and improved function [50]. Contraindications include unstable fractures or violations of the posterior vertebral body wall and middle column elements, thereby risking extravasation of cement into the spinal canal [51].

Polymethylmethacrylate (PMMA) is the most common cement of choice used in vertebroplasty (injection of cement into a collapsed vertebral body for the purpose of reinforcement) or kyphoplasty (percutaneous balloon inflation for restoration of height followed by cement augmentation) [52,53]. Alternatively, hydroxyapatite (HA) is a highly osteoconductive and biocompatible calcium phosphate-based material that can be used independently or administered in combination with other materials [54]. HA combined with PMMA can improve HA’s mineralization capacity and has been shown to have a lower risk of inducing thermal necrosis of adjacent structures during setting time compared to PMMA alone [55].

The 2011 CAFÉ study by Berenson et al. was a multi-center randomized controlled clinical trial that reported significant pain reduction 1 week after kyphoplasty in patients with painful vertebral compression fractures secondary to malignancy [56]. Patients reported a mean improvement of 3.8 points from the baseline score of 7.3 points, as measured by the numeric rating scale [56]. Notably, cement augmentation procedures do not require the use of general anesthesia, serving as a potential therapy for pain in cancer patients who are too unwell for other surgical interventions [51].

### 3.8. Fenestrated Screw Fixation

Poor bone quality, as seen in osteoporosis or metastatic disease, can pose a major challenge for patients who require stabilization for spinal metastases [51]. Pedicle screw fixation can effectively treat spinal fractures, deformities, and instability, all of which can result from spinal metastases [57]. Previously, the most common method of screw placement for spinal surgery with cement augmentation was injection of PMMA via cannula directly into the vertebral body, followed by placement of a non-fenestrated pedicle or lateral mass screw [57]. However, a frequent complication of using non-fenestrated pedicle screws is screw loosening after thoracolumbar stabilization, with a reported rate of 0.8% to 15% in non-osteoporotic patients treated with rigid systems [57,58]. Fenestrated pedicle screws with cement augmentation have been reported to improve pedicle screw anchorage in patients with osteoporosis and spinal metastases [59]. This is because fenestrated screws allow for the injection of cement after screw placement [60].

The nature of fenestrated pedicle screws, mainly the presence of an opening at the distal end of each screw through which cement is injected, poses a notable risk of cement extravasation, which has been shown to increase the likelihood of pulmonary cement embolism [61]. The risks of fenestrated pedicle screws are reflected in a 2021 retrospective study by Massaad et al. of 69 patients with spinal metastasis, which found that 502 cement-augmented fenestrated pedicle screws were placed in total [62]. The results show that no patients had screw failure, 145 of the 502 screws were associated with cement extravasation (most commonly via segmental veins), 1 patient with cement extravasation showed symptomatic thoracic radiculopathy treated with decompression, and 1 patient had asymptomatic pulmonary cement embolism [62]. Patient-Reported Outcomes Measurement Information System Pain Interference and Pain Intensity scores improved significantly from baseline at 3-month follow-up; thus, the authors concluded that fenestrated pedicle screws with cement augmentation are effective at spinal stabilization [62].

### 3.9. CFR-PEEK Instrumentation

Titanium hardware has long been the mainstay material of spinal instrumentation due to its stiffness, strength, and ease of use [63]. Due to titanium’s high density, titanium hardware imparts imaging artifacts on radiography, CT, and MRI, potentially impairing the evaluation of postoperative imaging and monitoring for recurrence or progression of spinal metastases [64].

Carbon-fiber-reinforced poly-ether-ether-ketone (CFR-PEEK) instrumentation was introduced as an alternative to titanium. Compared to titanium, CFR-PEEK is radiolucent and nonmagnetic, granting it superior radiographic qualities [65]. This is supported by findings from Ringel et al. demonstrating reduced artifacts on CT and MRI with CFR-PEEK instrumentation [65]. In the largest CFR-PEEK study to date, Alvarez-Breckenridge et al. showed how reduced artifacts can enhance postoperative imaging, as physicians were more easily able to detect local recurrence in 12 out of 69 patients, emphasizing how improved visualization can prevent the epidural spread of disease [3].

CFR-PEEK instrumentation has been shown to reduce the effective radiation dosage of tissue behind the hardware, a concept known as dose attenuation [66]. Nevelsky et al. reported less severe dose alteration with CFR-PEEK instrumentation compared to titanium, which decreases the possibility of under- and overdose of adjacent tissues [66]. While CFR-PEEK had 5% maximal dose perturbation, titanium resulted in 30% perturbation [66]. This is further corroborated by data from Poel et al. showing improved dose calculation accuracy with CFR-PEEK implants due to reduced imaging artifacts [67].

CFR-PEEK is a thermoplastic composite material with excellent biocompatibility, wear resistance, chemical/thermal resistance, and fracture toughness [3]. In terms of biomechanical stability, Cofano et al. demonstrated a non-inferior profile of CFR-PEEK when comparing the rates of intraoperative and postoperative complications, as well as the neurological and functional recovery of patients compared to titanium [68]. Cadaveric studies of biomechanical stability demonstrated similar mean bending and cycling capacity between CFR-PEEK and titanium instrumentation, resulting in comparable screw anchorage, resistance to motion, and stiffness [69,70]. However, CFR-PEEK’s degree of elasticity is more similar to that of cortical bone, resulting in less hardware failure due to better distribution of mechanical stress, subsequently decreasing the risk of mechanical complications [3,71]. A recent study in which six CFR-PEEK rods were retrieved from revision surgeries and evaluated for surface damage and internal changes, found that they demonstrated satisfactory biocompatibility, corrosion resistance, chemical stability, and mechanical properties and did not crack until 1.36 million fatigue tests; the junction between the nut and the rods was identified as a susceptible point of breakage, with the recommendation to secure nuts with a counter wrench and prevent overtightening [72]. An additional biomechanical benefit of CFR-PEEK is its reduced strain on adjacent vertebral levels, with a 2017 study by Kang et al. concluding that CFR-PEEK hardware significantly decreased the occurrence of adjacent-level degenerative disease and the risk of pedicle screw fracture compared to titanium [73].

However, CFR-PEEK instrumentation is not without disadvantages, impeding its routine implementation in spinal oncology. Mainly, concerns revolve around the high cost and low availability of CFR-PEEK instrumentation compared to titanium [32]. One strategy to mitigate the high cost is to use CFR-PEEK screws just above and below the area of interest and regular titanium pedicle screws for the remainder of longer constructs. CFR-PEEK implants can be difficult to visualize on fluoroscopy due to their radiolucency, and rods must typically be shaped preoperatively [74]. A study by Zavras et al. examining the opinions of neurosurgeons and orthopedic surgeons, suggests that surgeons believe CFR-PEEK-based implants are more difficult to manipulate and contour intraoperatively than titanium implants [63]. Other common themes included the lack of available options for cervical reconstruction, percutaneous options for MIS, and rod options with the ability to contour [63]. Lastly, CFR-PEEK hardware possesses lower tensile strength than titanium, which may contribute to implant failure [63].

In conclusion, CFR-PEEK instrumentation may enhance early disease detection, treatment visibility, and radiotherapy effectiveness. A review by Takayanagi et al. found the biostability and biocompatibility of CFR-PEEK to be similar to those of titanium [74]. Drawbacks to CFR-PEEK instrumentation include high costs, rod bending challenges, and limited intraoperative visibility [74]. Lastly, the radiolucency of CFR-PEEK instrumentation improves imaging clarity for detecting tumor recurrence, and its minimal dose perturbation enhances radiotherapy precision, offering better tumor control than titanium [74].

### 3.10. Radiofrequency Ablation

Radiofrequency ablation (RFA) involves using high-frequency alternating currents to denature malignant cells via ionic agitation [75]. Precise control over temperature and power is required, as heat deposition above 100 degrees Celsius could lead to tissue charring [75]. Therefore, a coaxial bipolar ablation probe can be used to automatically adjust the power to maintain heating within the desired treatment range [76]. The probe tip size determines the ablation volume, and larger tumors require more time to ablate [75]. Typically, RFA is conducted under general anesthesia or sedation under fluoroscopy or CT guidance [77].

RFA has been shown to provide significant local tumor control through the induction of coagulative necrosis in malignant cells [78,79]. Thus, a contraindication for RFA is significant coagulation impairment. Due to RFA’s potential to weaken healthy surrounding bone, it is typically used in conjunction with cement augmentation [80]. In a 2016 retrospective study of 55 spinal metastasis patients treated by RFA with cement augmentation, Wallace et al. found the one-year local tumor control rate to be 70% [79]. Beneficially, RFA has been shown not to interfere with adjuvant radiation or chemotherapy [79].

Pain palliation via RFA is achieved via multiple mechanisms: the destruction of pain-sensitive periosteum, decompression of nervous tissue, and removal of pro-inflammatory cytokines [80]. A 2016 prospective multicenter study by Bagla et al. investigated the impact of RFA with cement augmentation on the pain, disability, and quality of life of patients with vertebral body metastases [81]. They reported significant reductions in both pain and disability, as measured by the Numerical Pain Rating Scale (NPRS) (*p* < 0.0001) and Oswestry Disability Index (ODI) (*p* < 0.08) respectively [81]. There were no adverse events related to the RFA treatment [81]. These results were corroborated by Reyes et al., who demonstrated decreased Visual Analog Scale scores from 7.9 to 3.5 (<0.0001) and a reduction in ODI scores from 35.9 to 21.6 (*p* < 0.0001) post-procedure [82]. Again, no adverse events related to the RFA treatment were reported [82].

In a 2021 study by Abdelgawaad et al., RFA was combined with balloon kyphoplasty (BKP) for palliative treatment of neoplastic lesions [83]. Seventy of seventy-five metastases were treated with both transpedicular RFA and BKP, resulting in decreased post-procedure pain VAS scores (*p* = 0.0001), which illustrates the utility of combined RFA and BKP for painful spinal metastases [83]. No complications were reported due to RFA [83].

Potential side effects of RFA include local hematoma, hyperthermia, and an increased pain index during the immediate postoperative period [84,85]. More serious complications include superficial tissue burns and neurovascular injuries [86,87]. Tomasian et al. reported minor complication rates of 2.6% and major complication rates of 0.4% among 166 patients with RFA combined with vertebral augmentation [88]. Overall, the study concluded that RFA is safe for the treatment of spinal osseous metastases, with a 3.0% rate of complications [88].

RFA is a versatile and effective treatment option for patients with spinal metastases. Its minimally invasive nature, ability to provide pain palliation, tumor control, and compatibility with cement augmentation and radiotherapy make it a valuable therapeutic approach. Furthermore, RFA can be utilized as a standalone treatment, expanding its potential for improving outcomes.

### 3.11. Next Generation Sequencing

Precision medicine uses the sequenced genome to direct treatment. Extensive cancer genome characterization has led to an increasing discovery rate of cancer biomarkers. Next-generation sequencing (NGS) provides massively parallel sequencing and can address an extensive number of gene biomarkers with rapid speed, facilitating faster identification of genomic mutations [89]. As more oncogenic driver alterations are identified, the development of new targeted treatments specific to the genetic profile of the disease has the potential to completely alter the current landscape of cancer treatment.

Lung, breast, and prostate cancers are among the most prevalent neoplasms to metastasize to the spine, the treatment of which has been revolutionized by targeted therapy [90]. In targeted therapy, genomic data are used to identify actionable gene mutations that can be used to predict patient responsiveness to certain treatments [5]. An ideal example of targeted therapy is how the mutation status of EGFR can inform the treatment of non-small-cell lung cancer, which commonly metastasizes to the spine [91].

In lung cancer, patients with spinal metastases have a poor prognosis, with the overall median survival varying from 3.6 to 9 months [92]. EGFR mutation status is a crucial factor in determining the effectiveness of EGFR-tyrosine kinase inhibitors (TKIs), as activating mutations of EGFR tyrosine kinase domains can lead to further malignancy [91]. This knowledge was applied to clinical treatment in a 2019 study by Liang et al., which found that the irreversible inhibitor afatinib significantly improved progression-free survival and overall survival in patients with lung adenocarcinoma who possessed p.L747P and p.L747S EGFR mutations [93].

It is critical to highlight that as precision medicine becomes more routinely implemented, it is necessary to consider the methodological limitations of certain laboratory methods, including standard DNA sequencing or the use of commercial kits [93]. Liang et al. noted the potential for p.L747P and p.L747S EGFR mutations to be misclassified as a 19DEL mutation using the above methods due to poor DNA quality or less-than-ideal specimens lacking a large enough proportion of tumor cells [93]. In light of these results, they recommend the use of NGS to correctly detect these mutations to inform clinical decision-making [93]. The widespread application of NGS would enable physicians to capture more patients with p.L747P and p.L747S EGFR mutations and preferentially treat them with afatinib, leading to better patient outcomes [93].

With advancements in our knowledge of oncologic molecular markers, personalized treatment for metastatic spinal disease will likely improve. NGS provides comprehensive genomic information that can be used to find rare mutations to better target therapies. Future research, in particular, more robust studies on the efficacy of targeted therapy in the context of spinal metastases treatment, could make routine utilization of NGS in cancer management regimens a reality.

### 3.12. Artificial Intelligence

The field of AI comprises a collection of technologies with the potential to facilitate breakthroughs in the management of spinal metastatic disease, especially in imaging analysis and surgical planning [94]. The benefits of any proposed intervention will come at some cost, whether it be morbidity, invasiveness, recovery time, or financial burden, all of which must be weighed against the patient’s life expectancy, values, and potential for meaningful recovery. Therefore, AI can be used to help physicians generate patient-specific algorithms to inform their care without inherent human bias. These revolutionary technologies are already being applied to radiology, cancer diagnostics, neurosurgery, and oncologic treatment, where automated solutions offer increased reproducibility and precision. To enable proper implementation of the treatment plan, physicians should be knowledgeable about AI technologies and their applications to the management of spinal metastatic disease.

Knowing the basic differences between existing AI technologies will help physicians discern which technology to use for a particular purpose. ML is a statistical technique that attempts to fit models to data and then “learn” by training those models with data [94]. There are three categories of ML, supervised learning, semi-supervised learning, and unsupervised learning [95]. Supervised learning requires a training dataset for which the outcome variable of choice is already known and is the type of learning most commonly seen in clinical applications of AI [95]. There are two steps to supervised learning: training and testing [96]. During model training, training samples with their corresponding clinical labels are paired to train the model, and then they determine the relationship between the feature and the clinical labels [96]. In the testing phase, they use the trained model to evaluate its predictive performance [96]. It must be noted that supervised models often require large quantities of training samples to prevent overfitting [96].

In contrast, unsupervised learning does not use labeled training data but offers the potential to uncover new patterns or classifications from massive amounts of data by grouping data into clusters [95,96]. Unsupervised models utilize a distance measurement to calculate the similarity between samples [96]. Semi-supervised learning has aspects of both supervised and unsupervised learning. For example, semi-supervised learning first has an unsupervised feature learning phase, followed by a supervised model training phase [96]. Semi-supervised learning models use unlabeled data to mine tumor information, then utilize small amounts of labeled data to model the relationship between features and clinical labels [96].

Diving a level deeper, a neural network represents a more complex form of ML that views problems in terms of inputs, outputs, and the weights of “features” that associate inputs with outputs, allowing for categorization applications like predicting whether a patient will develop a particular disease [94]. Deep learning (DL) is the most complex form of ML, as it involves using neural network models with many levels of features that predict outcomes [94]. DL is increasingly being applied to radiomics, which involves extracting large amounts of quantitative features from medical imaging, combining imaging features with clinical and genomic information, and then mining these data to detect radiomic biomarkers [96]. For instance, Wang et al. applied a deep neural network to identify spinal metastases in MR images, which exhibited an accuracy of 90% in identifying spinal metastases, meeting clinical requirements [97]. Another study by Shi et al. studied the value of MRI-based radiomics in predicting the treatment response of breast cancer patients with vertebral metastases [98]. Wakabayashi et al. developed a radiomics model to predict pain response post-radiotherapy for spinal metastases [99].

ML has many applications in the field of precision medicine, a major one being the prediction of short-term mortality [100]. Karhade et al. created an ML algorithm to predict 30-day mortality after surgical intervention for spinal metastases. The 30-day mortality rate was found to be 8.49% for the 1790 patients [100]. The best preoperative predictors of 30-day mortality were low hematocrit and albumin levels, and high white blood cell count and alkaline phosphatase levels [100]. In addition, calibration measures demonstrated that the values predicted by the algorithm correlated well with the observed outcomes of the patients studied [100]. The limitations of this study included limited postoperative outcome data beyond 30 days and variation in patient and surgical characteristics in the used database [95].

The same analysis was then run again to find the best predictors of 90-day mortality (preoperative albumin, primary tumor histology, and Eastern Cooperative Oncology Group Performance Score) and 1-year mortality (preoperative albumin, preoperative hemoglobin, and tumor histology), publishing their findings as the SORG machine-learning algorithm, also referred to as the SORG Nomogram [101]. Multivariate analysis performed by Ahmed et al. in 2018 showed that the SORG Nomogram demonstrated the highest accuracy at predicting 30- and 90-day postoperative survival for metastatic spine disease [102].

Another survival prediction instrument is the New England Spine Metastasis Score (NESMS), which can predict 3-, 6-, and 12-month overall survival expectancy [103]. In a prospective comparison by Schoenfeld et al., NESMS was found to have a discriminative capacity significantly greater than that of the Tomita, Tokuhashi, and SINS scores, which means it was able to differentiate survival to a higher degree [104]. Table 1 summarizes the discussed survival prediction models.

Future directions of AI point towards generative AI, which has the ability to synthesize new data that can be used to train AI models for rare medical conditions [95]. The biggest advantage of generative AI-based data are that it allows for sharing across institutions without concerns about breaching patient confidentiality, as the data are synthetic. Down the line, generative models will allow for a better understanding of longitudinal multimodal patient data, as well as the incorporation of that data into predictive models to inform surgical planning [95].

As technology continues to evolve, the most crucial requirement for the further development of machine-based learning systems in modern medicine is the collection of high-quality data. The lack of current research on the topic of ML in spine oncology only speaks to the infancy of the topic. However, AI applications to spinal metastasis treatment such as the SORG Nomogram promise that future clinical decision-making will occur with even more accuracy and precision and less human bias than ever before.

## 4. Conclusions

As the treatment modalities for spinal metastatic tumors continue to evolve, the goal of improving the quality of life of patients with spinal metastases becomes more easily attainable. In this review article, we highlight various new treatment options that have shown promising improvements in patient outcomes. Notably, we discuss the potential clinical applications of AI and NGS in the treatment of spinal metastases.

The main findings reported in the literature for the most important considerations in the treatment of spinal metastases are summarized in Table 2. Considering what has been published in the literature, it seems reasonable that the best patient care is a combination of multiple treatment modalities, surgical and nonsurgical, that maximizes the advantages of each modality. Therefore, it is pertinent to remember that adjuvant therapies are equally fundamental to achieving greater pain reduction and improved patient outcomes in patients with spinal metastases. Lastly, the exact surgical and nonsurgical therapies used to treat patients with spinal metastases should be individualized according to their unique clinical presentation, pathology, and life expectancy.

## Figures and Tables

**Table 1:** A highlight of survival prediction models for metastatic spinal disease.

Prognostic Model	Year of Study	Design	Number of Patients	Statistical Method(s)
Tokuhashi	1990	Retrospective, Single Institution	64	Cox proportional hazard regression analysis
Tomita	2001	Retrospective, Single Institution	342	Cox proportional hazard regression analysis
Revised Tokuhasi	2005	Semi-prospective, Single Institution	246	Cox proportional hazard regression analysis
NESMS	2015	Retrospective, Multicentric Institutional	318	Cox proportional hazard regression analysis
SORG Normogram	2016	Retrospective, Single Institution	649	Regression normogram boosting algorithm

**Table 2:** Overview of studies on treatment modalities for metastatic spine tumors.

Study	Citation Number	Design	Year	Main Findings
I. Multidisciplinary Teams and Treatment Decision-Making Approach				
Rothrock et al.	[9]	Single-center retrospective review	2021	Survival rates have improved for patients with spinal metastases from kidney, breast, lung, and colon cancers, underscoring the importance of considering long-term outcomes in surgical treatment decisions.
Kimura	[18]	Systematic review	2018	A multidisciplinary specialist board approach to the management of bony metastases may improve quality of life and prognosis of patients by targeting reductions in morbidity, hospitalization rate, and overall costs associated with late-stage cancers.
Morgen et al.	[21]	Semi-prospective clinical study	2017	The original 2005 Tokuhashi prognostic scoring system for patients with metastatic spinal cord compression which evaluates factors such as performance status, neurological status, and the number of extraspinal bone metastases is improved upon in this updated scoring system.
El Saghir et al.	[19]	Narrative review	2014	Positive outcomes from tumor boards depend on the presence of qualified and effective faculty, good preparation and selection of cases, format and structure of the meeting, expertise, efficient leadership, and interactions among physicians present. Tumor boards allow for discussion, dissemination and implementation of guidelines, may help capture cases for clinical trials, and in areas with limited resources (such as rural hospitals), limitations in diagnosis and management can be overcome, or at least optimized.
Omlin et al.	[13]	Prospective case–control study	2013	Nutrition impact symptoms common to cancer patients such as pain, fatigue and taste and smell alterations influence cancer cachexia, which may in turn lead to compromised treatment outcomes. This may be countered via nutrition-targeted interventions.
Fisher et al.	[2]	Systematic review, modified Delphi technique	2010	The Spine Instability Neoplastic Score (SINS) classification system aids physicians in identifying when patients with neoplastic disease of the spine may benefit from surgical consultation. It can also aid surgeons in assessing the key components of neoplastic spinal instability and may become a prognostic tool for surgical decision-making when put in context with other key elements such as neurologic symptoms, extent of disease, prognosis, patient health factors, oncologic subtype, and radiosensitivity of the tumor.
Bilsky et al.	[22]	Systematic review	2006	The NOMS paradigm offers a comprehensive decision-making framework for treating spinal metastases, considering tumor radiation sensitivity, epidural extension, spinal stability, and systemic disease to determine optimal treatment strategies.
Patchell et al.	[8]	Multicenter, non-blinded RCT	2005	Survival advantage and superior ambulatory outcomes in patients with spinal metastases treated with a combination of surgery and radiotherapy compared to radiotherapy alone.
Massie	[16]	Systematic review	2004	Untreated depression results in significant morbidity and mortality. There is a higher prevalence of depression in certain cancer types (oropharyngeal, pancreatic, breast and lung). The spectrum of depression symptoms may vary through the course of cancer, as this patient population faces repeated threats to life, fluctuating pain levels, and more.
Tomita et al.	[20]	Retrospective case series	2001	A proposed patient-centered prognostic scoring system that provides weighted point-based evaluation of factors such as expected rate of tumor grown, visceral metastases, “treatable” vs. “untreatable”, bony metastases, and solitary vs. multiple lesions.
II. Surgery				
Iia. MIS				
Cui et al.	[24]	Single-center retrospective study	2021	Minimally invasive tubular surgery for spinal metastasis is safe and effective, particularly for patients with hypo-vascular tumors due to lower complicaiton rates.
Barzilai et al.	[23]	Prospective cohort study	2018	Minimally invasive surgery in spinal metastases reduces pain and symptom interference with daily activities.
IIb. Pre-op Embolization				
Damante et al.	[30]	Retrospective single-center review	2023	Preoperative embolization was associated with improved local control (LC) and pain outcomes in spinal metastatic patients.
Griessenauer	[31]	Meta-analysis and systematic review	2016	Embolization of spinal tumors is a safe and effective treatment option to reduce intraoperative blood loss, despite the variability in techniques and the retrospective nature of the available studies
Clausen et al.	[29]	Single-blind, randomized controlled clinical trial	2015	Intraoperative blood loss or the need for allogenic RBC infusions were not reduced with preoperative embolization in patients with spinal metastases. For hypervascular metastases, there was a small reduction in blood loss with pre-op embolization.
Kato et al.	[28]	Retrospective cohort study	2013	Reducing intraoperative blood loss by preoperative embolization was more effective when spinal metastases surgery was conducted on the same day compatred to later.
Kato et al.	[27]	Retrospective cohort study	2012	Preoperative embolization significantly reduced intraoperative blood loss, despite variations in tumor vascularization, embolization completeness, or timing of surgery relative to embolization
Owen	[26]	Systematic review	2010	Preoperative embolization of bone tumors is of specific benefit where there is a known high risk of bleeding during surgery, demonstrated spinal involvement and neural encroachment, where active bleeding is present, or in awkward surgical locations where prolonged surgery is anticipated.
IIc. Cement Augmentation				
Alsoof et al.	[53]	Systematic review	2022	Surgical interventions, such as kyphoplasty and vertebroplasty, provide superior pain relief compared to nonoperative management.
Koto et al.	[54]	in vivo experimental animal study	2017	Zoledronic acid-loaded bone cement reduced metastatic bony tumor growth of numerous cancer types in mice without causing systemic toxicity or adverse bone reactions.
Health Quality Ontario, Pron et al.	[51]	Systematic review	2016	Vertebroplasty and kyphoplasty effectively provide pain palliation and reduction in functional diabilities in cancer patients with vertebral compression fractures.
Berenson et al.	[56]	Multicenter randomized RCT	2011	Kyphoplasty is an effective, safe, and rapid treatment for painful vertebral compression fractures compared to non-surgical management.
IId. Fenestrated Screw Fixation				
Massaad et al.	[62]	Retrospective analysis	2022	Cement augmentation via fenestrated pedicle screws offers a reliable and effective method for spine stabilization in cancer patients. There is a minimal risk of significant adverse effects resulting from cement leakage.
Saadeh et al.	[59]	Systematic review	2020	Cement-augmented fenestrated pedicle screws enhance fixation strength in osteoporotic patients undergoing spinal fusion, reducing screw pullout and improving fusion rates compared to traditional techniques. Cement extravasation is a potential risk, but is most commonly asymptomatic.
IIe. CFR-PEEK (CFRP) Instrumentation				
Alvarez-Breckenridge et al.	[3]	Retrospective chart review	2023	Use of CFRP implants in spinal surgeries for oncologic patients and found them to be a safe and effective alternative to titanium implants resulting in minimal imaging artifacts, thereby facilitating postoperative radiation planning and the ability to detect local recurrence.
Zavras et al.	[63]	NASS Spinal Oncology Section survey	2022	Responders exhibited a lack of consensus with regard to the imaging and radiation benefits of radiolucent spinal implants, with concerns cited such as high costs, low availability, limited cervical and percutaneous options, and suboptimal screw and rod designs.
Poel et al.	[67]	Comparative experimental study	2020	CFRP implants reduce artifacts on CT images, decrease the time required for artifact correction, and lead to fewer discrepancies between planned and delivered radiation doses in proton therapy compared to titanium implants; CFRP may thereby improve outcomes for patients requiring both spinal stabilization and proton therapy.
Ringel et al.	[65]	Prospective observational study	2017	CFRP pedicle screws effectively reduce imaging artifacts on CT and MRI in comparison with standard titanium alloy implants, resulting in improved radiation planning.
Kang et al.	[72]	Comparative biomechanical analysis	2017	Titanium rods result in higher disk pressure and facet joint contact forces on adjacent segments compared to PEEK and CFRP rods. CFRP found to be superior to PEEK.
III. Radiation Therapy-EBRT and SRS				
Wong et al.	[36]	Meta-analysis and systematic review	2023	There is no significant difference in overall pain response between Stereotactic body radiation therapy (SBRT) and cEBRT. However, SBRT may provide better complete pain response at 3 and 6 months compared to cEBRT, without increasing the risk of adverse events. There were no significant differences in local progression and overall survival.
Gottumukkala et al.	[41]	Systematic review	2021	A review of the fundamentals of radiation oncology for treatment of spinal metastases, this article summarizes basic principles regarding treatment, complications, and the essentials of a multi-disciplinary approach to care. SBRT for spinal metastases reveal high rates of pain response and local tumor control, including cases in which reirradiation is indicated.
Kumar et al.	[32]	Systematic review	2020	Wound complications post spinal metastases surgeries are significantly associated with the timing of radiation therapy (RT). Postop-RT has fewer wound complications versus preop-RT. Evidence is insufficient to recommend a precise ideal RT to surgery interval. However, an interval of 2 weeks with the minimum being 7 days is optimum.
Barzilai et al.	[37]	Review article	2017	An updated review integrating the most recent decade of evidence-based medicine for treatment of spinal metastasis into the NOMS framework. The most important change to these paradigms has been the integration of SRS, allowing delivery of tumoricidal radiation doses with sparing of nearby organs at risk High-dose single or hypofractionated SRS offers a significantly higher biologic effective dose and more precise dose delivery to the tumor with shorter treatment schedules compared with the cEBRT. Integration of SRS has fundamentally changed the indications for and type of surgery performed for metastatic spine tumors.
Bate et al.	[40]	Retrospective chart review	2015	SRS provides durable local disease control while preserving or improving neurological function either alone (if separation surgery is not necessary) or as an adjunt to surgical decompression. This is true even for historically radioresistant tumor types such as renal cell carcinoma.
Witham et al.	[38]	Review article	2006	RT has a clearly defined role for treatment of metastatic tumors of the spine with epidural compression; this is especially true for radiation-sensitive tumors in the setting of non-bony spinal cord compression and those with a limited life expectancy. Spinal stereotactic radiosurgery, vertebroplasty and kyphoplasty are emerging treatment options that are beginning to be used in selected patients with metastatic spinal tumors with epidural compression.
Wong et al.	[36]	Meta-analysis and systematic review	2023	There is no significant difference in overall pain response between Stereotactic body radiation therapy (SBRT) and cEBRT. However, SBRT may provide better complete pain response at 3 and 6 months compared to cEBRT, without increasing the risk of adverse events. There were no significant differences in local progression and overall survival.
IV. Radiofrequency Ablation (RFA)				
Wallace et al.	[78]	Retrospective cohort study	2016	RFA in combination with vertebral augmentation is effective for local spine metastases control. Further, it does not interfere with administration or effectiveness of radiation or chemotherapy.
Bagla et al.	[80]	Multicenter prospeective clinical series	2016	RFA with concurrent vertebral augmentation is safe and effective in providing quick pain relief, disability reduction and improvemnt in quality of life for patients with vertebral body metastases.
V. Artificial Intelligence and Next Generation Sequencing (NGS)				
Wang et al.	[96]	Original research with exploratory data analysis	2017	A multi-resolution approach using deep Siamese neural networks was developed to identify spinal metastases in MRI images, exhibiting an accuracy rate of 90%.
Wakabayashi et al.	[98]	Original research with exploratory data analysis	2021	A model using a combination of clinical and radiomic features to predict the pain response of patients with spinal metastases receiving radiation therapy. The sensitivity and specificity of the combined features model were 85.4% and 76.2%.
Karhade et al.	[99]	Original research with exploratory data analysis	2019	A machine learning (ML) algorithm was developed to predict 30-day mortality after surgical intervention for spinal metastases. The 30-day mortality rate was found to be 8.49% for 1790 patients. An open access web application was developed for the best performing model.
Ahmed et al.	[101]	Retrospective study	2018	A comparison of the ability of scoring systems to estimate both overall survival (OS) at various time points and tumor-specific survival for patients undergoing surgical treatment for metastatic spine disease. The the Skeletal Oncology Research Group (SORG) Normogram demonstrated the highest accuracy at predicting 30-day and 90-day survival after surgery. The original Tokuhashi was the most accurate at predicting 365-day survival.
Schoenfeld et al.	[102]	Observational study with exploratory data analysis	2021	The New England Spinal Metastasis Score (NESMS) is validated as a useful prognostic tool for predicting survival in patients with spinal metastases regardless of of selected treatment strategy. The NESMS may be used in patient care, hospital-based practice and health care policy.
Barzilai et al.	[5]	Retrospective study	2022	NGS data sourced form spinal metastases demonstrated a high concordance rate for genetic alterations between the primary tumor and spinal metastasis. This was also true between spinal metastases and other, visceral metastases, especially for driver mutations. Therefore, spine tumor samples may be reliably used for genomic-based decision-making in cancer care, particularly for prostate, NSCLC, and breast cancer.
Cao et al.	[90]	Retrospective cohort study	2023	Multiparameter MRI-associated radiomics ML models were developed to predict the presence of the epidermal growth factor receptor (EGFR) mutation and subtypes sourced from the spinal metastasis in patients with pathologically confirmed primary lung adenocarcinoma. The models integrating T1 and T2FS sequences achieved the best prediction capabilities.
